# Ferrite Film Loaded Frequency Selective Metamaterials for Sub-GHz Applications

**DOI:** 10.3390/ma9121009

**Published:** 2016-12-13

**Authors:** Bo Gao, Matthew M. F. Yuen, Terry Ye

**Affiliations:** 1 Department of Mechanical Engineering, Hong Kong University of Science and Technology, Clear Water Bay, Kowloon 999077, Hong Kong; meymf@ust.hk; 2 SYSU-CMU Joint Institute of Engineering, SYSU-CMU International Joint Research Institute, Foshan 528000, China; terryye@andrew.cmu.edu

**Keywords:** metamaterials, split ring resonators, ferrite film

## Abstract

Electromagnetic metamaterials are constructed with sub-wavelength structures that exhibit particular electromagnetic properties under a certain frequency range. Because the form-factor of the substructures has to be comparable to the wavelength of the operating frequency, few papers have discussed the metamaterials under GHz frequency. In this paper, we developed an innovative method to reduce the resonant frequency of metamaterals. By integrating the meta-structures with ferrite materials of higher permeability, the cell size of the meta-structure can be scaled down. This paper describes the methodology, design, and development of low-profile GHz ferrite loaded metamaterials. A ferrite film with a permeability of 20 could reduce the resonant frequency of metamaterials by up to 50%. A prototype has been fabricated and the measurement data align well with the simulation results. Because of the lowered operational frequency, the proposed ferrite loaded metamaterials offer more flexibility for various sub-GHz microwave applications, such as cloaks, absorbers, and frequency selective surfaces.

## 1. Introduction

During the past few decades, Metamaterials have been the focus of many research studies as a new substance to build novel electromagnetic and electronic devices. Benefitting from their artificially engineered repetitive structures, metamaterials can exhibit special properties with both negative permittivity and permeability under a certain frequency range. These properties make metamaterials ideal for antenna enhancement and optimization, especially for antennas used in non-electromagnetic-friendly ambient conditions and surroundings. Metamaterial structures were first introduced by Veselago in 1968 [1,2,3]. They consists of ordered repetitive structures called a “periodic unit cell” or “cell”. The cell structure, differing in the shape, arrangement, and geometry, manipulates the electromagnetic wave propagation inside the metamaterials. Just like atoms or molecules of natural materials, the structure of the repetitive unit cells determines the properties of the metamaterials. More specifically, the electromagnetic performance of metamaterials can be tuned by modifying those unit cells, by adding high dielectric material loading, multilayer metamaterial design, and filling curve designs [4,5,6,7,8,9,10,11,12,13,14,15,16,17,18,19,20,21,22,23].

In order to achieve their unique electromagnetic properties, the feature size of cells inside most metamaterials is designed to be around the sub wavelength scale of the frequency under operation, such that the meta-structure resonates with the electromagnetic wave. This feature size is practically feasible for applications under optical or high frequency microwave ranges, i.e., several hundred nanometers for optical devices and several millimeters at the 10–20 GHz range. However, when it comes to sub-GHz applications, the feature size is scaled up to the 100 mm range. With IoT (Internet of Things) applications gradually emerging and succeeding the Internet in the new millennium, the sub-GHz and sub-10 GHz frequency wireless communications, i.e., channels at 150 MHz, 315 MHz, 433 MHz, 900 MHz, and 2.4 GHz dominate IoT applications [24]. Many of the applications, such as RFID (Radio Frequency Identification) and sensor devices, are attached to objects with non-electromagnetic-friendly materials, or need compact but higher-gain antennas for embedded installation. Therefore, metamaterials for the sub-GHz frequencies are of great importance. To meet the IoT wireless communication requirements, ferrite based metamaterials have been proposed in the last few years [25,26,27,28,29,30]. Most of the ferrite based metamaterials use Yttrium iron garnet (YIG) rods or permanent magnetic ferrite rods combining with metal wire however, the cost of ferrite rods is high and they are difficult to fabricate. In this paper, we exploit the ferrite film, which is made of low-cost dispersed ferrite particles on cured polymer substrate, and integrate it with a Split-Ring Resonator (SRR) as the meta-structure cell. The structure has a smaller feature size, with a resonant frequency lowered by almost 50% compared with the structures without ferrite film loading. The proposed metamaterial is ideal for sub-GHz IoT applications.

## 2. Design of SRR Metamaterials

**Figure 1:**
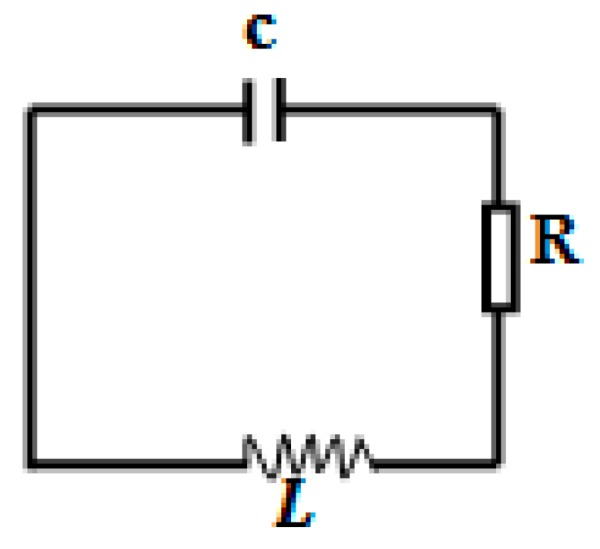
Equivalent circuit model of the SRR. C is the intrinsic capacitance. L is the intrinsic inductance. R is the intrinsic resistance which introduces the system loss.

The SRR (split ring resonator) is one of the most studied metamaterials. The structure exhibits strong magnetic coupling to an electromagnetic field. A SRR metamaterial consists of repetitive cells of an enclosed metal loop with splits at the ends. In addition to the negative refractive index property, SRR metamaterials are also widely used as a frequency selective structure to reflect, transmit, or absorb electromagnetic waves operating at the resonant frequency. SRR metamaterials are usually fabricated on printed circuit board (PCB) with a 2D planar repetitive structure. The split ring resonators consist of two rings in the unit cell. The equivalent circuit model of a SRR is shown in Figure 1, where C is the intrinsic capacitance, L is the intrinsic inductance, and R is the intrinsic resistance which represents the system loss. The impedance of the metamaterial surface (assume an infinite 2-D plane) for a perpendicular incident electromagnetic wave is

(1) $$Z=\frac{j\omega L}{1-\omega^{2}\text{LC}}$$

where Z is the impedance of surface, ω is the frequency of incident electromagnetic wave, L is the sheet inductance, and C is the sheet capacitance. The surface impedance becomes infinite when the frequency reaches its resonant frequency.

(2) $$\omega_{0}=\frac{1}{2\pi \sqrt{\text{LC}}}$$

**Figure 2:**
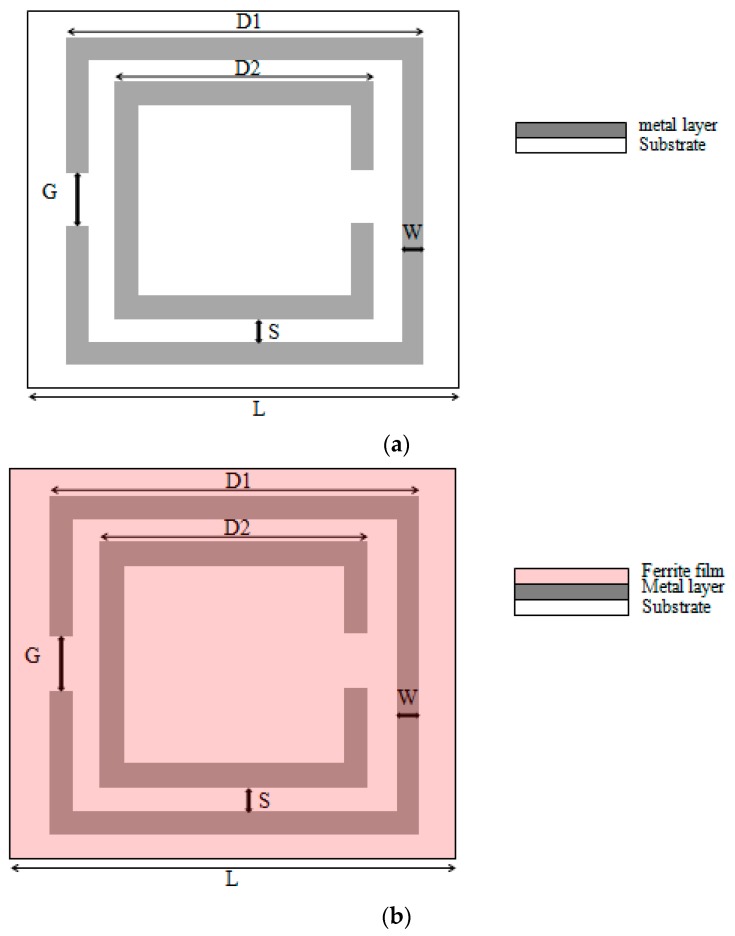
Schematics of the split ring resonator (SRR). (**a**) The metal conductive traces are on the dielectric substrate. The unit cell size is 25 mm × 25 mm; (**b**) The metal conductive traces are on the dielectric substrate. Another layer of ferrite film is attached on the metal layer. The unit cell size is 25 mm × 25 mm. D1: outer length of the SRR; D2: inner length of the SRR; G: split width in the rings; W: width of the ring trace; S: distance between the rings; L: unit cell size; t: thickness of the substrate; ε: The permittivity of the substrate.

The design of the SRR is shown in Figure 2 with the dimension of the structure. D1, D2, G, W, S, L, t, and ε are 22 mm, 15 mm, 3 mm, 2 mm, 1.5 mm, 25 mm, 1.6 mm, and 4.4, respectively. Although the SRR structure is an ideal frequency selective resonator for metamaterials, the size of a traditional SRR is determined by the designated resonant frequency, and the size of the structure could be impractically too large for GHz applications. For the designs in Section 2, the 25 mm × 25 mm unit cell SRR has a resonant frequency at 2.68 GHz. If we want to achieve a sub-GHz resonant frequency, the unit cell size needs to be enlarged to about 60 mm, which is quite impractical for GHz microwave applications. For example, the UHF RFID tag (Ultra High Frequency Radio Frequency Identification) operates from 860 to 960 MHz. The tag size is usually 50–70 mm in diameter. A 60 mm unit cell size is too big to be used as a RFID performance booster.

In this paper, we developed an innovative and low cost method to lower the resonant frequency of SRR metamaterials. A layer of commercially available ferrite film with a higher permeability and permittivity was used. The ferrite film is made of ferrite particles mixed in a polymer matrix with a permeability of 20 and permittivity of 10. The composition of this ferrite film is shown in Table 1. As shown in Figure 1, the resonant frequency of SRR depends on the intrinsic inductance and capacitance. The addition of a ferrite film layer increases the SRR's capacitance and inductance simultaneously. The high capacitance and inductance reduces the resonant frequency, thus tuning the SRR structure’s resonant frequency to the GHz and sub-GHz range.

To demonstrate the enhancement of the ferrite film, we designed two metamaterials with SRR structures for comparison, i.e., one loaded with the above mentioned ferrite film, and the other without. Figure 2 shows the design of the proposed SRR. A layer of ferrite film of 0.3 mm was placed on top of the SRR surface. The two metamaterial structures were analyzed in the finite element method software—ANSYS HFSS. The unit cell of the designed metamaterials was simulated with periodic boundary conditions in a finite element model. The transmission and reflection of this unit cell were simulated to acquire the transmission parameters.

**Figure 3:**
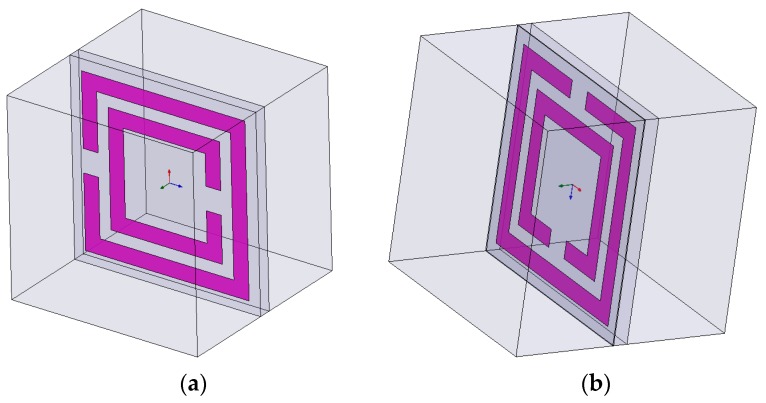
Simulation setup of (**a**) unit cell of the SRR. Two wave port excitation was placed on the left and right side to excite the SRR unit; (**b**) unit cell of SRR-Ferrite. A layer of ferrite film of 0.3 mm was placed on the top of the SRR surface. The ferrite film with a permittivity of 10 and permeability of 20 could increase the intrinsic inductance of the SRR unit cell.

**Figure 4:**
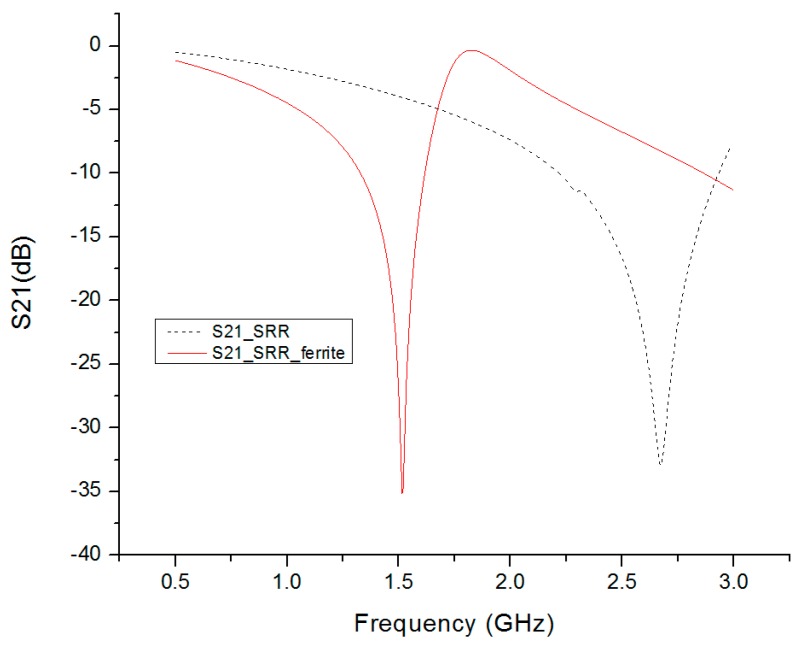
Simulation results of the S21 parameter of the SRR unit cell and SRR-ferrite unit cell. The resonant frequency of the SRR unit cell was about 2.68 GHz. For the SRR unit cell loaded by ferrite, the resonant frequency was about 1.5 GHz.

As mentioned, the SRR is defined as a periodic and infinite plate. Periodic boundary conditions were used to simulate an infinite plate. In this case, the perfect electric (E) boundary and perfect magnetic (H) boundary were used as periodic boundaries, as shown in Figure 3. A perfect E boundary was used to represent a perfectly conducting surface in the SRR structure. The electric field was assumed to be normal to the surface of the SRR. A perfect H boundary represents a surface on which the tangential component of the H-field is the same on both sides. For surfaces on the outer surface of the model, this resulted in a boundary that simulates a perfect magnetic conductor in which the tangential component of the H-field was zero. Two wave ports were also assigned to excite this SRR structure. This wave port is defined as a waveguide with the same cross section of the port. Wave ports were placed on the left and right interfaces to provide a window that couples the model device to the external environment. The conductive traces were defined as a 2D copper sheet without thickness. The conductivity was defined as 58,000,000 siemens/m, which is the conductivity of copper. This simulation was swept from 0 to 3 GHz and the simulation results are shown in Figure 4.

For the design without ferrite film loading, the resonant frequency of this SRR was around 2.68 GHz, where the S21 (transmission) was a minimum. The operating frequency of the Ferrite film loaded SRR was 1.5 GHz, as shown in Figure 4. This demonstrated that the resonant frequency of metamaterials could be significantly reduced by adding a layer of ferrite film. The resonant frequency was decreased by about 45% by adding a layer of 0.3 mm thick ferrite film, with 20 permeability and 10 permittivity.

## 3. Prototype Preparation and Measurement Results

**Figure 5:**
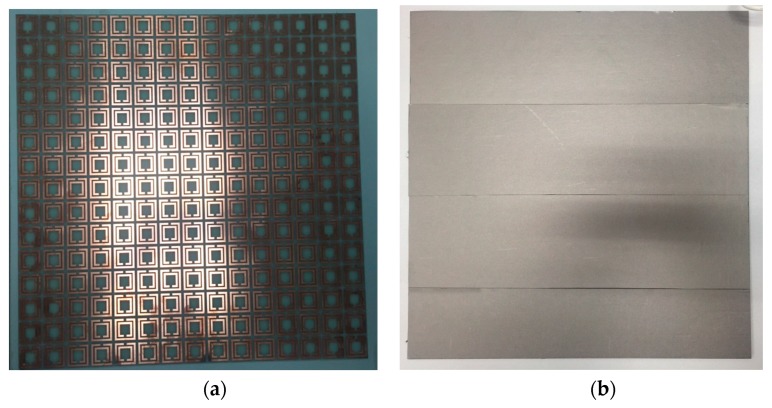
Prototypes of metamaterials with and without ferrite film. (**a**) The picture of a slab of 15 mm × 15 mm units of SRR metamaterials; (**b**) the 15 mm × 15 mm units SRR metamaterial slab covered by a layer of 0.3 mm thick ferrite film.

The metamaterial with the SRR cell was fabricated on a PCB board, where the dielectric material was FR4 with a dielectric constant of 4.4. Two prototypes were fabricated for comparison; One with a layer of 0.3 mm thick ferrite film attached on the surface of the SRR metamaterial, as shown in Figure 5. The film covered the whole area of the SRR metamaterial. The other prototype had no ferrite film, but had an identical SRR structure. A repetitive array of 15 mm× 15 mm SRR unit cells were fabricated on the FR4 substrate. The size of the thin slab was 375 mm × 375 mm × 1.6 mm. The conductive copper trace thickness was 35 um.

**Figure 6:**
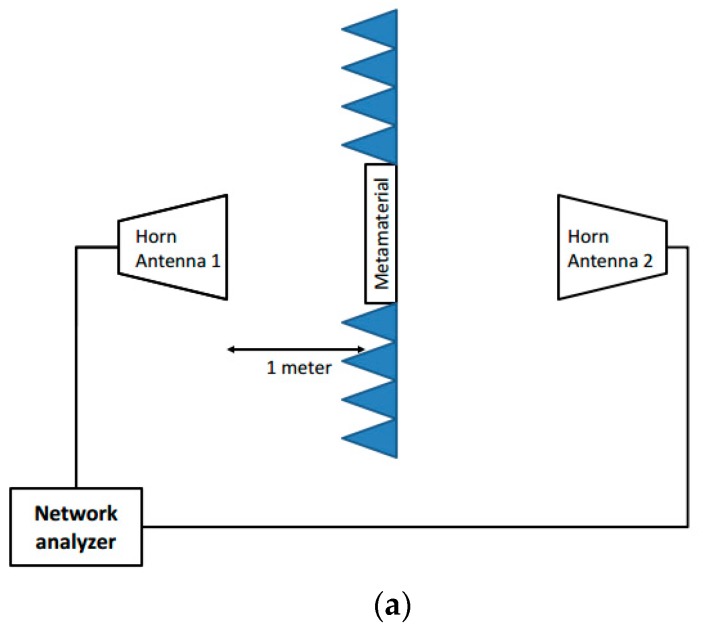

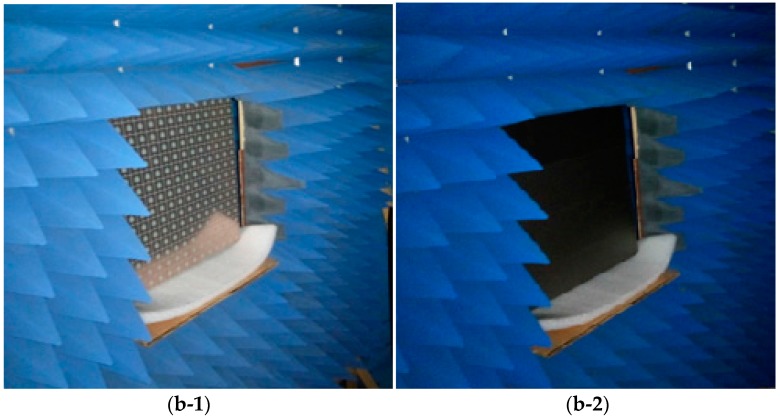
Measurement setup (**a**) schematic of the measurement setup. The metamaterial is surrounded by microwave absorbers. Two horn antennas operating from 0.8 to 8.0 GHz are used to transmit and receive signals. The network analyzer connects the two horn antennas. The distance between the metamaterial and both horn antennas is 1 m. Photo of the test setup. The measurement is conducted in an anechoic chamber to eliminate external measurement noise. (**b-1**) the SRR slab and (**b-2**) the SRR covered by 0.3 mm thick ferrite film.

**Figure 7:**
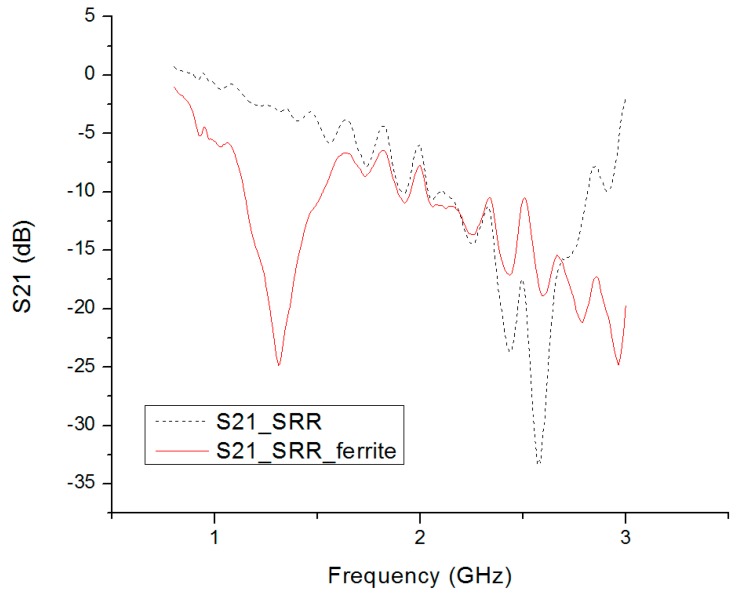
Measurement results of the SRR slab and SRR slab covered by a 0.3 mm thick ferrite film. The resonant frequency of the SRR slab only was around 2.58 GHz. The resonant frequency of the ferrite covered SRR was 1.5 GHz.

In HFSS simulation, the metamaterial property was extracted from the S parameter of the SRR cell. The measurement was performed in a 3 m (width) × 3 m (height) × 3 m (length) anechoic chamber, with microwave materials covering the wall surface. A horn antenna was used in the transmission testing to retrieve the S21 parameter. The layout of the measurement setup is shown in Figure 6a. Two other horn antennas operating from 0.8 to 8 GHz were used. One was used as a transmitter and the other was used as a receiver. The antennas were placed 1 m away from the metamaterial to make sure the incident wave was a planar wave. The testing structure was supported by a foam stand as shown in Figure 6b-1,b-2. The system was calibrated before the test to eliminate the systematic effects. The transmission parameter measurement excluded the influence of the transmission path. For S21 calibration, the absence of metamaterial was used as the normal transmission calibration. The measurement results are shown in Figure 7. The resonant frequency of the SRR slab without ferrite film loading was 2.58 GHz and the resonant frequency of SRR covered by ferrite film was 1.3 GHz. The measurement results demonstrated that the ferrite film decreased the resonant frequency by about 50%.

The results from the simulation and prototype measurement align quite well. The comparison between the simulation and measurement is listed in Table 2. There is only a couple of tens of MHz of resonant frequency difference. This difference might be introduced by discrepancies between the product specifications and samples during prototype fabrication. In simulation, the material parameter was FR4 taken from the HFSS library. The properties of the prototype, especially the ferrite film, might be different from the datasheet and library. Second, the conducting material used in the simulation was 2D planar copper. The material thickness was not considered, which might also introduce errors. The fabrication of the samples might also have introduced errors. Last, the permeability of this composite will change with the frequency. The permeability value is 20 at 1 GHz. Since this paper focuses on the application of metamaterials for the sub-GHz frequency range, this permeability value at 1 GHz was set as a reference value. To save simulation time, we choose this value for all frequencies in HFSS. This might also have introduced differences between the simulation and measurement results.

## 4. Discussion and Conclusions

In this paper, we first demonstrated an innovative approach to lower the resonant frequency of metamaterials to the sub-GHz range by using a ferrite film layer on a SRR structure. Compared with other approaches using ferrite rod which is expensive and difficult to fabricate, the ferrite film used in this paper is a low cost film and is widely used for EMC/EMI (Electromagnetic Compatibility/Electromagnetic Interference) and other applications. The measurement results showed that the resonant frequency could be reduced by up to 50% by applying this ferrite film. It is believed that this kind of ferrite film loaded metamaterials could be widely adopted in sub-GHz IoT applications, such as cloaks and absorbers. The ferrite film loaded metamaterials can be thin and flexible. The flexible nature of the ferrite film also makes the metamaterials suitable for non-flat surfaces; this make it possible to develop flexible cloaks and absorbers.

## Figures and Tables

**Table 1:** Composition percentage of ferrite film [31].

Substance	Weight Percentage (wt %)
Iron	65–85
Silicon	2–7
Aluminum	3–8
PU (Polyurethane)	10–20
Additive	1–3

**Table 2:** Comparison of the scattering parameters (S parameters). The resonant frequencies of SRR and SRR-ferrite are listed. The measured resonant frequency was lower than the simulation results.

Name	Resonant Frequency (Simulation)	Resonant Frequency (Measurement)	Bandwidth (Simulation)	Bandwidth (Measurement)
SRR	2.68 GHz	2.58 GHz	0.26 GHz	0.51 GHz
SRR-ferrite	1.5 GHz	1.3 GHz	0.39 GHz	0.78 GHz
